# Effects of Seedling Age on Colonization Patterns of *Citrus limon* Plants by Endophytic *Beauveria bassiana* and *Metarhizium anisopliae* and Their Influence on Seedlings Growth

**DOI:** 10.3390/jof6010029

**Published:** 2020-02-25

**Authors:** Bamisope Steve Bamisile, Komivi Senyo Akutse, Chandra Kanta Dash, Muhammad Qasim, Luis Carlos Ramos Aguila, Hafiza Javaria Ashraf, Wei Huang, Mubasher Hussain, Shiman Chen, Liande Wang

**Affiliations:** 1 State Key Laboratory of Ecological Pest Control for Fujian and Taiwan Crops, College of Plant Protection, Fujian Agriculture and Forestry University, Fuzhou 350002, China; bamisopebamisile@yahoo.com (B.S.B.); luisramosaguila@gmail.com (L.C.R.A.); 1151903001@m.fafu.edu.cn (H.J.A.); huangwei2450@163.com (W.H.); shimanchen08@163.com (S.C.); 2 Plant Health Theme, International Centre of Insect Physiology and Ecology, P.O. Box 30772, Nairobi 00100, Kenya; 3 Department of Entomology, Faculty of Agriculture, Sylhet Agricultural University, Sylhet 3300, Bangladesh; dashck.entom@sau.ac.bd; 4 Ministry of Agriculture Key Lab of Molecular Biology of Crop Pathogens and Insects, Institute of Insect Science, Zhejiang University, Hangzhou 310058, China; cmqasimgill@zju.edu.cn; 5 Plant Protection College, Fujian Agriculture and Forestry University, Fuzhou 350002, China; 6 Institute of Applied Ecology and Research Centre for Biodiversity and Eco-Safety, Fujian Agriculture and Forestry University, Fuzhou 350002, China; 7 Guangdong Key Laboratory of Animal Conservation and Resource Utilization, Guangdong Public Laboratory of Wild Animal Conservation and Utilization, Guangdong Institute of Applied Biological Resources, Guangdong Engineering Research Center for Mineral oil pesticides, Guangzhou 510260, China; mubasherhussain05uaf@yahoo.com

**Keywords:** fungal endophytes, artificial inoculation, colonization frequency, fungal entomopathogens, foliar spraying

## Abstract

The inoculation methods, the fungal strains, and several other factors are known to influent the success of fungal entomopathogens colonization in plants. The physiological status of the plant could also be another determinant. In the present study, the ability of three strains of *Beauveria bassiana* and one strain of *Metarhizium anisopliae* to successfully colonize *Citrus limon* plants and the influence of seedling age on endophytic colonization success was examined. Three, 4, and 6 months old seedlings were inoculated with 10 mL of 1 × 10^8^ conidial·mL^−1^ suspensions of each of the four fungal strains via foliar spraying. All fungal strains successfully colonized citrus seedlings and were sustained up to 2 months in colonized plants irrespective of the seedling age, with differences in the mean percentage colonization recorded at various post-inoculation periods among the fungal strains. The highest percent endophytic fungi recovery rate was recorded in the 3 months old seedlings, where fungal mycelia of inoculated fungi were successfully re-isolated from 65.6% of the untreated newly developed leaf and stem tissues. One strain of *B. bassiana*, BB Fafu-12, significantly improved seedling height and leaf number. The study demonstrates the influence of seedling age on *B. bassiana* and *M. anisopliae* successful colonization in the citrus plant.

## 1. Introduction

For many decades, chemical insecticides have been the most commonly used method for suppressing insect pests [1]. However, persistent use of these insecticides has resulted in the development of resistance by pests, posing environmental and economic hazards, as well as exposing users to various health challenges [2]. Recently, entomopathogenic fungi have been adopted as an alternative control measure to replace chemical pesticides [3]. Entomopathogenic fungi have been reported to infect several species of crop pests such as aphids, thrips, mites, and other economic insects. *Beauveria bassiana*, for instance, has been effectively used to control several insect pests, usually through innundative applications [4,5]. Notable examples are *Diaprepes* citrus root weevil (*Diaprepes abbreviates*), black citrus aphid (*Toxoptera citricida*), citrus longhorn beetle (*Anoplophora chinensis*), glassy-winged sharpshooter (*Homalodisca vitripennis*), and the Asian citrus psyllid (*Diaphorina citri*) [3,6,7]. However, aside from foliar spraying of targeted pests or plants with fungal entomopathogens suspension to offer short term protection for plants against pests infestation [7], a practice which reduces the efficacy of entomopathogenic fungi due to the exposure of fungal spores to environmental conditions such as UV radiation, high temperature, low humidity, etc. [8], previous findings have revealed the possibility of establishing these entomopathogenic fungal species as endophytes in plants [9,10,11,12,13]. Endophytic fungi can function as plant growth-promoting agents, exhibiting important changes in the development and yield of several crops [14,15,16,17,18]. A typical example is *B. bassiana*, which has a wide-ranging host plant species, hence, a worldwide distribution, making it the most abundant species of the numerous entomopathogenic fungal species reported to date [19]. *Metarhizium anisopliae*, on the other hand, is not as common in nature as *B. bassiana*. However, the fungus has also been reported as a plant tissue colonizer, plant growth enhancer, or as a naturally occurring endophyte [15,20,21,22,23].

Artificial inoculation of plants with entomopathogenic fungi and their establishment as endophytes has been successfully demonstrated in several arable crops such as corn [24,25,26], common bean [11,18,27], wheat [10,26], tomato [28,29], sweet pepper [5], sorghum [30], opium poppy [31,32], and in some other economically important crops. However, aside from the reports of *B. bassiana* in cocoa by Posada and Vega [33]; Posada et al. [34] *B. bassiana* in radiata pine by Brownbridge et al. [35], *Lecanicillium* sp. and *B. bassiana* in date palm by Gómez-Vidal et al. [36], *B. bassiana* in coffee by Posada et al. [37], *Clonostachys rosea* in oak tree by Kwaśna et al. [38], and *B. bassiana* in horse-chestnut trees by Barta [39], studies on artificial inoculation of entomopathogenic fungi in tree crops are still scanty. *Citrus limon* and the rest of other citrus species are fruit crops of huge economic importance around the world in lieu of their popularity, availability, nutritional value, contribution to foreign exchange, and industrialization [40]. The prospect of entomopathogenic fungi becoming endophytic in citrus plants would assist future studies to design pest management programs that could mitigate attacks caused to citrus seedlings and trees by the various citrus plant pests; such as the Asian citrus psyllid (*D. citri*) and its transmitted disease pathogen—*Candidatus* Liberibacter asiaticus; the causal organism of citrus greening disease, which constitute a serious threat to citrus production across the globe [40].

Several factors have been reported as influencing the success of establishing entomopathogenic fungi as endophytes in the host plants. The growth media used for raising the plant [30], the inoculation methods used [26,41,42], the species of the plant, the fungal species, and inoculum density [11,37] have all been identified as potential colonization promoters or impediments. The age of the plant is also another factor to be considered prior to artificial inoculation with entomopathogenic fungi, as the physiological status of the plant might also influence the success of endophytic colonization.

Elucidating the influence of seedling age on the outcome of entomopathogenic fungi colonization in citrus plants would guide future studies in the selection of seedlings for fungal inoculation trials. In the current study, greenhouse experiments were conducted to (1) assess entomopathogenic fungal strains of the genera *Beauveria* and *Metarhizium* for their potential to become endophytic in *C. limon*, (2) correlate the citrus seedlings age with the level of endophytic colonization success, (3) evaluate the level of endophytic colonization persistency over a period of time, (4) examine the prospects of the inoculated fungi to migrate from treated plant parts to untreated or newly emerged plant parts, and (5) evaluate the potential plant growth-enhancing effects of the inoculated fungal entomopathogens.

## 2. Materials and Methods

### 2.1. Source of Fungal Strains

The 3 fungal strains of *B. bassiana* (BB Fafu-12, BB Fafu-13, and BB Fafu-16) and 1 strain of *M. anisopliae* (MA Fafu-1) used in this study were obtained from the fungal culture bank of Fujian Agriculture and Forestry University (FAFU), Fuzhou, Fujian, China PR. BB Fafu-12 and BB Fafu-13 were isolated from mycosed adults of *Diaphorina citri,* while BB Fafu-16 and MA Fafu-1 were isolated from soil samples collected from different private citrus orchards around *Rixi*, a satellite town of Fuzhou, Fujian province, South Eastern part of China (26°21’8.88´´N, 119°16´9.52´´E). Fungal strains were selected for this study based on the outcome of preliminary studies conducted in the laboratory prior to the current study. The sequence data of the 4 fungal strains used in the present study have been deposited in the NCBI GenBank database. The accession numbers for BB Fafu-12, BB Fafu-13, BB Fafu-16, and MA Fafu-1 were MG844429, MG844430, MG844431, and MG844433, respectively.

### 2.2. Citrus Plants

Citrus seedlings (*Citrus limon* var. Sichuan *an yue*) used in the study were raised from surface-sterilized seeds washed in 70% ethanol for 3 min and 2% sodium hypochlorite for 3 min and then rinsed thrice in sterile distilled water. To confirm the success of the surface sterilization procedure, 100 μL of the final rinsed water was plated on potato dextrose agar media (PDA, Qingdao Hope Bio-Technology Co., Ltd., Qingdao, China) and incubated at 25 °C for 10 days. Following surface sterilization, seeds were sown in pollen trays containing sterilized compost, vermiculture, and garden soil mixes and transplanted at 40 days post-planting into 12 cm × 12 cm × 10 cm seedling pots filled with sterilized compost, garden soil, and vermiculture at 5:4:1 ratio. Prior to seed propagation, soil mixes were sterilized in an autoclave—Boxun YXQ-LS-75S (Shanghai Boxun Medical Biological Instrument Corp., Shanghai, China) at 120 °C for 2 h. This was done to remove existing microorganisms that might be present in the soil. The soil was thereafter left to cool for 72 h before use. Transplants were kept in a glasshouse at 25 °C (± 2 °C) and 65–75% relative humidity (RH), under a 12 h light: 12 h dark (L:D) photoperiod. Seedlings were watered at 5–6 days interval with sterile distilled water and fertilized monthly using 1 g·L^−1^ Basfoliar 30-10-10 + Mg + TE (COMPO Expert GmbH, Krefeld, Germany) inorganic fertilizer.

### 2.3. Conidial Suspension Preparation

Fungal cultures used in the study were grown on PDA. After fungal cultures preparation, plates were stored at 25 °C in an incubator. The conidia used for seedlings inoculation were harvested from 14–16 day-old cultures for *B. bassiana* (BB Fafu-12, BB Fafu-13, and BB Fafu-16) and 21–24 day old cultures for *M. anisopliae* (MA Fafu-1). The conidia were harvested by scraping the fungal colonies from the surface of the agar using a sterile scalpel under axenic conditions. Afterward, the harvested conidia were then suspended in 20 mL 0.01% Tween 80 solution, vortexed for 3 min, and then filtered using a sterile syringe and cotton wool to remove hyphal debris and obtain a clean stock suspension. To estimate the conidial concentration of the stock, 10,000 fold serial dilution of the stock suspension was prepared to enable conidia count, where 100 μL of the stock suspension was diluted with 900 μL of sterile water containing 0.01% Tween 80 solution, and then vortexed for 20 s; and the step was repeated for the next dilution. The inoculum was prepared by adjusting the initial stock concentration to a final concentration of 1 × 10^8^ conidia mL^−1^ using the Neubauer hemocytometer.

Prior to inoculation, the percentage of conidia viability was determined by plating 100 μL of the first serial dilution on 2.5% water agar and then incubated at 25 °C for 24 h. The viability was assessed by counting the number of germinated conidia. A conidium was considered to be viable when it develops and projects a germ tube that was longer than half of its diameter. The conidia suspension could only be used for inoculation when the average conidia germination percentage was above 90%.

### 2.4. Inoculation of Citrus Plants

The experiment was set up in a completely randomized design comprising 3 sampling groups (3, 4, and 6 months old seedlings), 4 treatments, and a control, each treatment with 15 replicates. The leaves of the plants were sprayed with 10 mL of 1 × 10^8^ conidial mL^−1^ suspensions of each fungal strain or with sterile distilled water containing 0.01% Tween 80 solution as a control. The soil surface was covered with aluminum foil to prevent conidia runoff. At the inoculation date, the inoculated leaves were counted and marked, while the plant height was measured. This was done to differentiate between the plant parts that were directly sprayed with conidia and the parts that emerged post-inoculation. In order to conserve humidity, the inoculated seedlings were covered with cellophane bags for 24 h. Treated seedlings were kept at 25 (± 2 °C) and 65–75% relative humidity (RH), under a 12 h light: 12 h dark (L:D) photoperiod until assessment for endophytic colonization was done.

### 2.5. Determination of Fungi Inoculation Effects on Plant Growth

To evaluate the effect of inoculated fungal entomopathogens on citrus seedlings growth, the height (the distance from plant base to the tip of the stem in cm) and leaf number of 5 randomly selected seedlings per fungal treatment and control were assessed at 7 days interval, up to 56 days post-inoculation (dpi). At each endophytic colonization assessment date, 5 seedlings were selected per treatment and control for plant growth assessment, 4 of which were sampled per treatment for endophytic colonization assessment using a destructive sampling method.

### 2.6. Endophytic Colonization Assessment

To evaluate the effects of plant age on endophytic colonization, leaf, and stem tissues of the same age on differently aged plants (3, 4, and 6 months old seedlings) were selected for endophytic colonization assessment. Individual seedlings were divided into 5 segments, that is: Upper leaves (UL) and upper stems (US), which were young/newly developed (untreated) plant parts that emerged after inoculation; lower leaves (LL) and lower stems (LS), which were older (treated) plant parts that were directly sprayed with fungal conidia suspension at inoculation date, and the roots. The endophytic colonization assessment was conducted at 7, 14, and 56 dpi. The assessment was done using a destructive sampling method, where plant tissues (i.e., leaves, stems, and roots) were cut into small sizes of about 4 cm length and then surface sterilized using 70% ethanol for 3 min and 2% sodium hypochlorite for another 3 min, and then rinsed thrice in sterile distilled water. The plant tissues were placed to dry on a sterile tissue paper. The outer edges of the surface-sterilized plant parts were trimmed off as the endophytes in this region might have been eliminated during surface sterilization. The plant tissues were further trimmed into smaller sections or pieces of about 8 × 8 mm long and plated on a 9.0 cm Petri dish containing freshly prepared PDA. Streptomycin sulfate and chloramphenicol at 1.25 g·L^−1^ were added to the medium to suppress bacterial contamination, while 5 plant tissues were placed in each Petri dish, sealed with parafilm, and incubated at 25 °C. To determine the success of the surface sterilization procedure, 100 μL of the last rinsed water was plated on agar and a tissue imprint was done, then incubated at 25 °C for 10 days. The corresponding samples were not considered for analysis if fungal growth was recorded from the Petri dish on which the last rinsed water was plated. The Petri dishes containing the plant tissues were also incubated at 25 °C in the incubator and inspected periodically at 2–3 days interval to assess them for fungal growth. Plant sections that exhibited fungal growth were recorded, and the fungus isolated and transferred into freshly prepared PDA plates to prevent contamination. Emerging fungal colonies recorded from plated plant tissues were morphologically identified by comparing the mycelia and growth pattern with the mother culture and by viewing the conidia and conidiophores through slides preparation using a light microscope (Model CX21FS1—Olympus Corporation, Tokyo, Japan) in line with Humber (1997, 2012).

### 2.7. Statistical Analyses

Before analysis, data were subjected to normality and homogeneity test of variances using qqplot, and Shapiro-Wilk Normality test (at 0.05 significance level). In order to stabilize the variance, colonization percentage data were subjected to log transformation before any statistical analysis was done. The Generalized Linear Method (GLM) up to a 3-way interaction was used for analyzing data of plant colonization by entomopathogenic fungi. Data on plant growth assessment were subjected to a two-way ANOVA. When a significant *F* test was obtained at *p* < 0.05, a multiple comparison was performed using the least significant difference (LSD) test. The colonization frequency of each fungal strain per plant segment was determined using the formula of Petrini and Fisher [43] as modified by Posada et al. [37] and Klieber and Reineke [44] as: Colonization frequency = 100 × (number of plants from which fungal endophyte was re-isolated/total number of plants treated with fungal entomopathogens). Data were analyzed using IBM SPSS statistical software v22.0 (SPSS Inc., Chicago, IL, USA) and Statistix 8.1 (Analytical Software, Tallahassee, FL, USA).

## 3. Results

### 3.1. Assessment of Endophytic Colonization in Inoculated Citrus Seedlings

**Figure 1:**
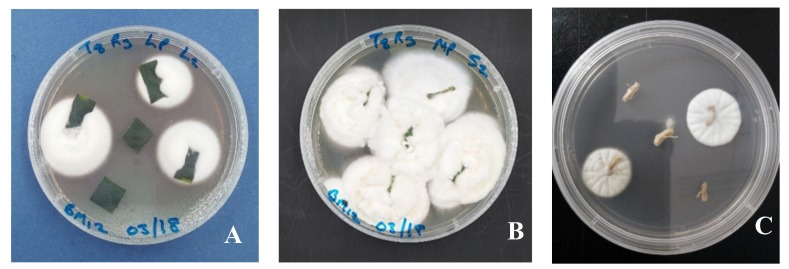
Endophytic *Beauveria bassiana* (BB Fafu-12) re-isolation from the citrus plant tissues. (**A**) Leaf, (**B**) stem, and (**C**) root tissues following foliar treatment with fungal conidia suspension.

All entomopathogenic fungal strains examined in this study (BB Fafu-12, BB Fafu-13, BB Fafu-16, and MA Fafu-1) were successfully established as endophytes in citrus seedlings following foliar inoculation across all three seedling-age ranges (3, 4, and 6 months post-planting). The successful re-isolation of inoculated fungal mycelia from surface-sterilized plant tissues plated on PDA confirmed the success of the colonization trial (Figure 1). The tissue imprints and the last rinsed distilled water used for sterilizing the plant tissues prior to tissues plating on PDA did not yield any fungal growth, hence confirming that the surface sterilization technique was effective. In addition, neither *B. bassiana* nor *M. anisopliae* was re-isolated from the control seedlings.

In terms of the overall performance of individual fungal strains across the three seedling-age ranges, BB Fafu-12 was the most successful of all the strains examined (F_4,88_ = 79.71, *p* < 0.001). With reference to the plant organs that were mostly colonized by the treated entomopathogenic fungi across all the various ages of seedlings, the highest endophytic fungi recovery rate was recorded in the leaves, which was significantly higher than the stems, while the roots were poorly colonized (F_2,88_ = 138.9, *p* < 0.001). Out of the four fungal strains evaluated, only BB Fafu-12 was recovered from the root tissues of 3 and 4 months old seedlings, but never from the root tissues of 6 months old seedlings (Table 1). All the other strains failed to colonize the root in all the seedling-age ranges. There was a significant interaction among treatments and the plant organs (F_8,88_ = 17.10, *p* < 0.001). Across all the seedling-age ranges, a significant decline in percentage colonization was observed with an increase in post-inoculation time (F_2,88_ = 6.17, *p* < 0.001). However, all the fungal strains that succeeded in colonizing the inoculated citrus seedlings were sustained up to 56 days post-inoculation in the colonized plants (Table 1). 

### 3.2. Effects of Seedling Age on the Success of Endophytic Fungal Entomopathogens Colonization of Citrus Plants

The highest mean percentage colonization was recorded in seedlings treated at 3 months post-planting compared to 4 and 6 months old seedlings (F_2,88_ = 20.99, *p* < 0.001). When the colonization rate of all tested fungal strains in combination was assessed in individual plant organs, significant differences among the seedling-age ranges were observed in the leaf (F_2,28_ = 6.87, *p* < 0.001), and stem (F_2,28_ = 16.51, *p* < 0.001), but not in the root (F_2,28_ = 1.34, *p* = 0.2776). There was a significant interaction among the age of seedlings and plant organs (F_4,88_ = 4.18, *p* < 0.001), however, the interaction among the treatments, the age of the seedling, and plant organs was not significant (F_16,88_ = 1.65, *p* = 0.0735). Regardless of the age of seedlings, the foliar parts of inoculated seedlings were readily colonized by all fungal strains assessed (Table 1).

### 3.3. Effect of Endophytic Fungal Entomopathogens on Seedlings Height and Leaf Number

**Figure 2:**
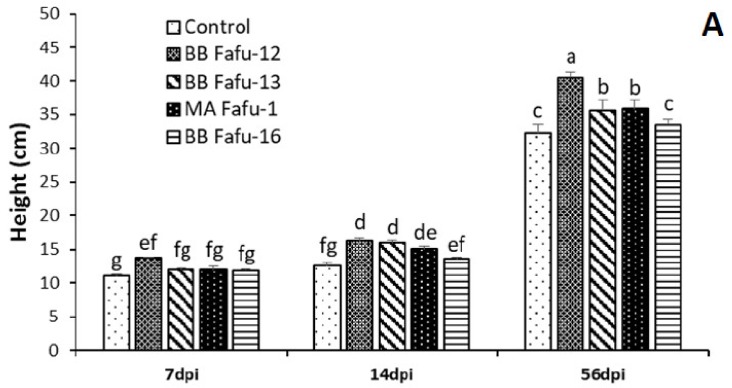

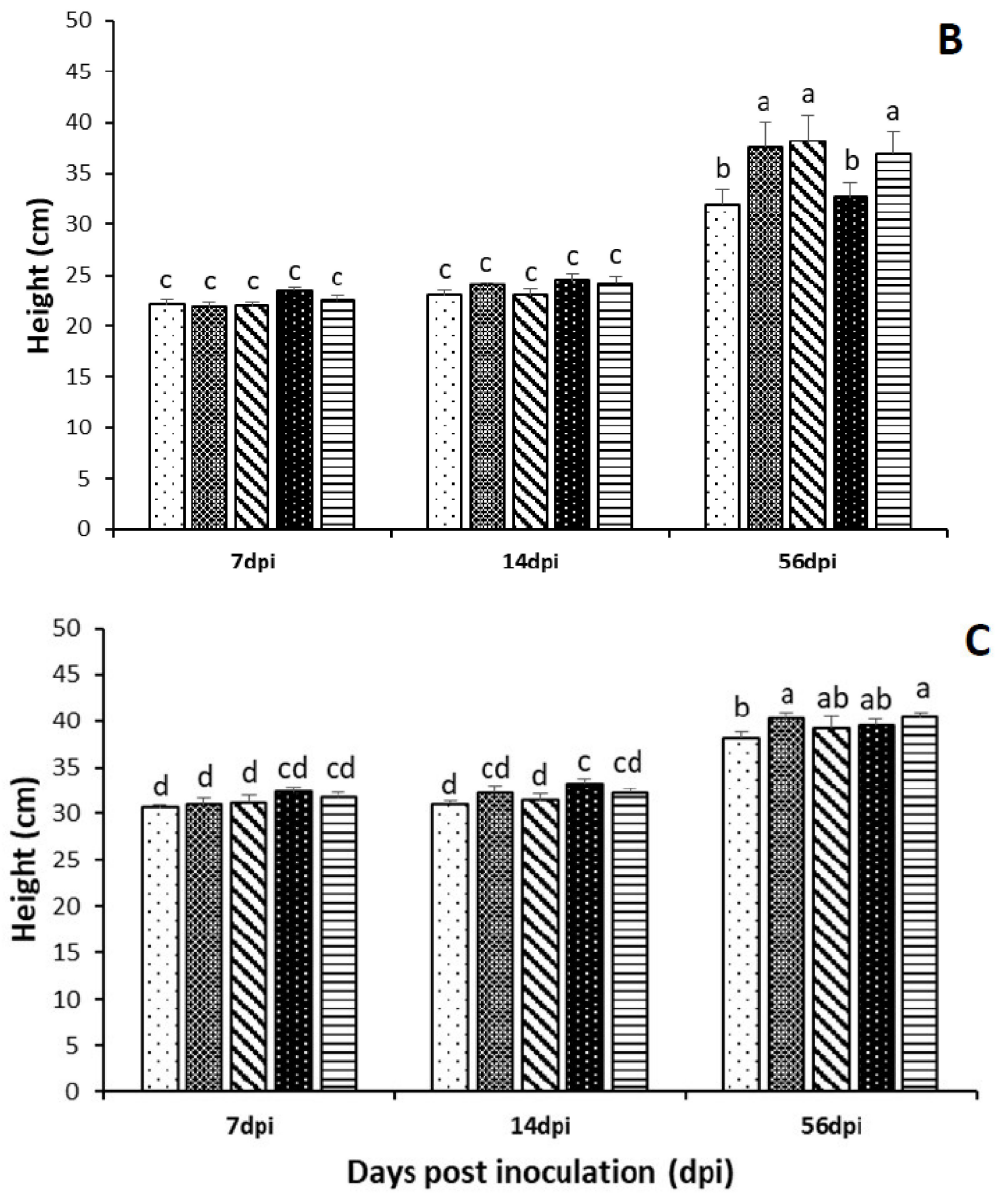
Mean (± SE) plant height of citrus seedlings at 7, 14, and 56 days post-inoculation (dpi) with entomopathogenic fungal strains (BB Fafu-12, BB Fafu-13, MA Fafu-1, and BB Fafu-16) or sterile distilled water containing 0.1% Tween 80 (Control). (**A**–**C**) are showing the height of citrus seedlings treated at 3, 4, and 6 months post-planting, respectively. Bars (± SE) with different letters indicate significant differences among treatments at *p* < 0.05 (LSD after a two-way ANOVA).

**Figure 3:**
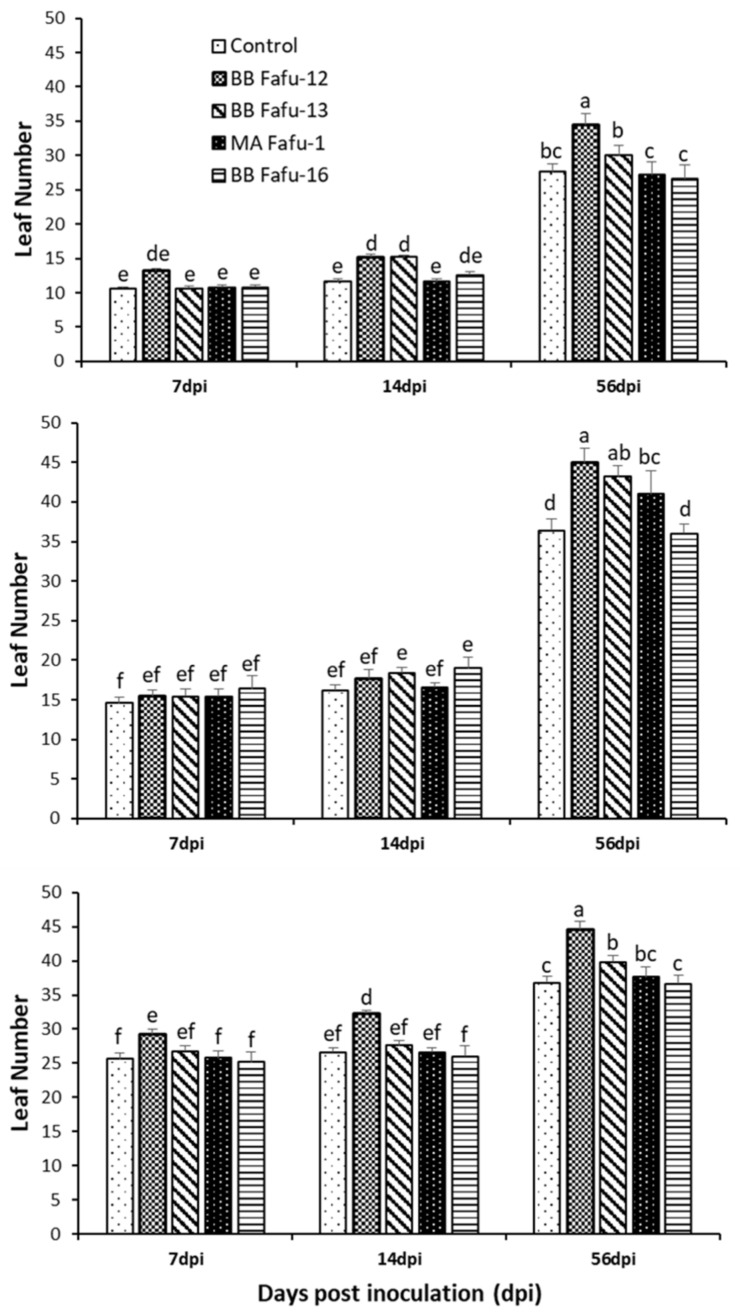
Mean (± SE) leaf number of citrus seedlings at 7, 14, and 56 days post-inoculation (dpi) with entomopathogenic fungal strains (BB Fafu-12, BB Fafu-13, MA Fafu-1, and BB Fafu-16) or sterile distilled water containing 0.1% Tween 80 (Control). (**A**–**C**) are showing the mean leaf number of citrus seedlings treated at 3, 4, and 6 months post-planting, respectively. Bars (± SE) with different letters indicate significant differences among treatments at *p* < 0.05. (LSD after a two-way ANOVA).

The seedling growth promotion assessment was done at 7, 14, and 56 dpi. In general, a significant improvement in the height of citrus seedlings colonized by *B. bassiana* was recorded in the 3 (F_8,56_ = 8.08, *p* = 0.0059; Figure 2A), and 4 months old seedlings (F_8,56_ = 2.22, *p* = 0.0394; Figure 2B), but not in the 6 months old seedlings (F_8,56_ = 0.58, *p* = 0.7928; Figure 2C). Similarly, the highest number of leaves were recorded in seedlings treated with BB Fafu-12 in the 3 (F_8,56_ = 2.09, *p* = 0.0502; Figure 3A) and 4 months old seedlings (F_8,56_ = 2.74, *p* = 0.0126; Figure 3B), which were significantly different from the control seedlings. 

### 3.4. Assessment of Systemic Endophytic Colonization Migration within the Citrus Seedlings by Entomopathogenic Fungi

Fungal treated citrus seedlings were examined for possible migration of fungal mycelia in-vivo, from plant parts that were directly sprayed with fungal conidia at inoculation date to other plant tissues that emerged post-inoculation. The mycelia of fungal strains that were successful in colonizing the seedlings were observed to migrate from treated plant parts to untreated newly emerged plant tissues. All *B. bassiana* strains (BB Fafu-12, 13, and 16) were readily re-isolated from the upper leaves (UL) and stems (US) of treated seedlings. However, the migration of *M. anisopliae* MA Fafu-1 was only limited to the leaf part, as no fungal recovery was recorded in any of the newly emerged stems of MA Fafu-1 treated seedlings in all seedling-age ranges (Table 2). Overall, inoculated conidia appeared to migrate better in younger seedlings, as 65.6% of both the upper leaves and stems of the 3 months old seedlings were colonized, as compared to 18.8% and 25% of the upper leaves and stems that were, respectively, colonized in 6 months old seedlings, while 46.9% of the upper leaves and 37.5% of the upper stems were colonized in four months old seedlings. In addition, fungal mycelia were re-isolated from 15.6% and 9.4% of the root tissues of 3 and 4 months old seedlings, respectively, whereas none was recovered from the root tissues of 6 months old seedlings (Table 2). 

## 4. Discussion

All the tested fungal strains, including the sole strain of *M. anisopliae,* were successful in colonizing citrus seedlings in all of the seedling age ranges. Although all of the four fungal strains showed the capacity to colonize citrus plants systemically, differences among the strains were observed. This outcome validates previous reports that have indicated dissimilarity between the two fungal species examined in this study with reference to the patterns of endophytism exhibited following their artificial inoculation in crop plants [23,45]. For instance, *B. bassiana* has been reported to have the potential of colonizing the entire plant, irrespective of the inoculation method used [22,27,29,30,35,37,46]. *M. anisopliae,* on the other hand, has been found to be preferentially localized in the rhizosphere [20], or mainly in the root tissues [15,21,23]. The variation in percent colonization among fungal strains may be due to their differential growth rate and endophytic abilities [27,47].

The ability of *B. bassiana* to colonize the roots, untreated newly formed leaves, and stem tissues of treated citrus plants showed that endophytes can indeed be translocated from inoculated regions to the entire plant. Bing and Lewis [48] gave an explanation to this as the endophyte might have been translocated together with photosynthates to other plant parts. The outcome of our study is similar to the report of Resquín-Romero et al. [49], where the leaves, stems, and roots of tomato, alfalfa, and melon plants were colonized following foliar treatment with *B. bassiana*. Qayyum et al. [29] also recorded similar observations in tomato plants treated with *B. bassiana*, where the whole plant was colonized following fungal treatment with different artificial inoculation methods. Endophytic *B. bassiana* has been observed in previous studies to directly penetrate the plant epidermis into the leaf internal organs, moving upwards and downwards from the point of inoculation systemically colonizing the entire plant through intercellular spaces and vascular xylem elements [25,31].

Similarly, the systemic colonization of *C. limon* by *M. anisopliae* that was found in this study supports the findings of Batta [50]. In the study, the author reported the recovery of the inoculated fungus from distant leaves, other than the sole leaf that was sprayed with fungal conidia, stems, and petioles, which confirmed the potential of *M. anisopliae* to move across the interconnected vascular tissues of oilseed rape plant. A similar outcome was reported in tomato plants following the application of *M. anisopliae* via the soil drenching method [51]. However, these findings are contrary to the reports of Behie et al. [22] and Greenfield et al. [23], which reported preferential localization of *M. anisopliae* in the root tissues of haricot beans and cassava, respectively. The plant species inoculated, the growth media, and the fungal strains used in various studies are the likely factors responsible for these contrasting findings [11,37].

The sustenance of *B. bassiana* and *M. anisopliae* up to 2 months in citrus plants supported previous studies that have reported prolonged sustenance of endophytic colonization in woody plants. Similar results have previously been demonstrated by Brownbridge et al. [35], where *B. bassiana* was sustained in radiata pine for 9 months. Similarly, *B. bassiana* was found to be sustained in the root tissues of coffee plants up to 6 months following stem injection with fungal conidia suspension [37]. The higher percentage endophytic recovery and fungal mycelia migration that was recorded in the younger seedlings showed that successful establishment and the effects of the fungi decrease with increasing plant age. This outcome might be due to the fact that the physiology of plants changes with age [52].

The present study also provides evidence of positive plant growth-enhancing effects in response to the treatment of plants with entomopathogenic fungi. Similarly, Kabaluk and Ericsson [53] reported improvement in the yield of field corn following treatment with *M. anisopliae*. Similar observation was recorded in tomato plants treated with *M. anisopliae*, where treated plants showed significant improvement in height, shoot dry weight, root length, and root dry weight [51]. Improvement in the stand counts and yield of wheat plants treated with *B. bassiana* was also reported by Reddy et al. [54]. The ability of endophytic fungi to induce the production of plant growth regulators is believed to be related to plant growth improvement. Entomopathogenic fungi endophytes have been reported to synthesize the growth hormones; auxins and cytokinins in colonized plants [55].

## 5. Conclusions

In summary, the successful establishment and sustenance of *B. bassiana* and *M. anisopliae* as fungal endophytes in *C. limon* plants provide an alternative management strategy to the conventional practice of inundating spraying of biopesticides in tree crops and against insect pests of citrus. The influence of plant age on endophytic colonization outcome, as demonstrated in the current study, also broadens the available data on endophytic colonization determinants. Aside from the inoculation methods, growth media and fungal strains to be used, the age of the seedlings should also be considered when selecting plants for entomopathogenic fungi inoculation trials. Although the underlying mechanisms behind the higher endophytic fungi recovery incidence and translocation that was recorded in younger plant tissues are still unclear and require further elucidation. Future studies would be focused not only on answering this question, but also to assess the pathogenicity of the endophytically-colonized citrus plants on major citrus pests.

## Figures and Tables

**Table 1:** Mean (± SE) percent colonization of different plant parts (leaf, stem, and root) of *Citrus limon* treated with fungal entomopathogens (*Beauveria bassiana* and *Metarhizium anisopliae*) at different seedling ages (3, 4, and 6 months old seedlings).

Fungal Strains	dpi	Colonization (%)								
		3 Months Old Seedlings			4 Months Old Seedlings			6 Months Old Seedlings		
		Leaf	Stem	Root	Leaf	Stem	Root	Leaf	Stem	Root
BB Fafu-12	7	88 ± 2.73 ^Aa^	80 ± 5.22 ^Aab^	0 ± 0.00 ^Bc^	62 ± 13.63 ^Aab^	46 ± 17.46 ^Ab^	0 ± 0.00 ^Bc^	70 ± 3.78 ^Aab^	50 ± 3.78 ^Ab^	0 ± 0.00 ^c^
	14	52 ± 14.24 ^Bab^	68 ± 4.91 ^ABa^	25 ± 7.04 ^Abc^	38 ± 9.32 ^ABCabc^	48 ± 13.88 ^Aab^	0 ± 0.00 ^Bc^	40 ± 15.12 ^ABCDabc^	28 ± 11.30 ^ABabc^	0 ± 0.00 ^c^
	56	50 ± 4.46 ^Ba^	40 ± 4.38 ^CDab^	16 ± 6.29 ^Abc^	32 ± 9.20 ^ABCab^	42 ±9.59 ^Aab^	20 ± 5.34 ^Aabc^	38 ± 8.81 ^ABCDab^	25 ± 9.06 ^ABabc^	0 ± 0.00 ^c^
BB Fafu-13	7	42 ± 5.45 ^Bab^	38 ± 2.73 ^CDEab^	0 ± 0.00 ^Bc^	50 ± 11.78 ^ABa^	25 ± 10.45 ^ABabc^	0 ± 0.00 ^Bc^	45 ± 11.18 ^ABab^	15 ± 9.82 ^Bbc^	0 ± 0.00 ^c^
	14	44 ± 8.88 ^Ba^	46 ± 4.18 ^BCa^	0 ± 0.00 ^Bc^	35 ± 8.59 ^ABCab^	15 ± 9.68 ^ABabc^	0 ± 0.00 ^Bc^	42 ± 13.33 ^ABCa^	8 ± 5.26 ^Bbc^	0 ± 0.00 ^c^
	56	38 ± 4.17 ^Ba^	34 ± 6.30 ^CDEa^	0 ± 0.00 ^Bb^	35 ± 9.82 ^ABCa^	10 ± 6.55 ^ABb^	0 ± 0.00 ^Bb^	3 ± 2.50 ^CDb^	3 ± 2.50 ^Bb^	0 ± 0.00 ^b^
MA Fafu-1	7	30 ± 8.18 ^BCab^	12 ± 8.18 ^DEFbc^	0 ± 0.00 ^Bc^	34 ± 6.29 ^ABCa^	0 ± 0.00 ^Bc^	0 ± 0.00 ^Bc^	10 ± 3.78 ^BCDbc^	0 ± 0.00 ^Bc^	0 ± 0.00 ^c^
	14	32 ± 10.17 ^BCa^	12 ± 8.77 ^DEFab^	0 ± 0.00 ^Bb^	30 ± 11.68 ^ABCa^	0 ± 0.00 ^Bb^	0 ± 0.00 ^Bb^	18 ± 8.81 ^BCDab^	0 ± 0.00 ^Bb^	0 ± 0.00 ^b^
	56	27 ± 6.25 ^BCa^	10 ± 5.39 ^EFab^	0 ± 0.00 ^Bb^	25 ± 7.32 ^ABCa^	0 ± 0.00 ^Bb^	0 ± 0.00 ^Bb^	10 ± 6.55 ^BCDab^	0 ± 0.00 ^Bb^	0 ± 0.00 ^b^
BB Fafu-16	7	38 ± 8.18 ^Bab^	34 ± 8.90 ^CDEab^	0 ± 0.00 ^Bb^	34 ± 8.90 ^ABCab^	42 ± 5.47 ^Aa^	0 ± 0.00 ^Bb^	35 ± 15.47 ^ABCDab^	20 ± 13.09 ^Bab^	0 ± 0.00 ^b^
	14	27 ± 8.30 ^BCab^	34 ± 6.30 ^CDEa^	0 ± 0.00 ^Bc^	16 ± 9.45 ^BCabc^	35 ± 8.59 ^ABa^	0 ± 0.00 ^Bc^	18 ± 7.96 ^BCDabc^	8 ± 3.66 ^Bbc^	0 ± 0.00 ^c^
	56	23 ± 8.30 ^BCab^	32 ± 7.99 ^CDEa^	0 ± 0.00 ^Bb^	15 ± 7.32 ^BCab^	32 ± 9.21 ^ABa^	0 ± 0.00 ^Bb^	15 ± 7.32 ^BCDab^	0 ± 0.00 ^Bb^	0 ± 0.00 ^b^

Colonization was assessed at 7, 14, and 56 days post-inoculation (dpi). Means (± SE) followed by different uppercase letters within the same column (that is; same seedling age and organ) or lowercase letters within the same row (that is; same dpi and fungal strain) indicate significant differences among treatments and seedling-age ranges respectively at *p* < 0.05. (least significant difference (LSD) after generalized linear method (GLM)). Data for control were not represented in the table as no fungi recovery was recorded in the untreated control seedlings.

**Table 2:** Colonization frequency of entomopathogenic *Beauveria bassiana* strains (BB Fafu-12, BB Fafu-13, and BB Fafu-16) and *Metarhizium anisopliae* (MA Fafu-1) in different plant segments (leaf, stem, and root) following foliar inoculation of 3, 4, and 6 months old citrus plants with 1 × 10^8^ conidia mL^−1^ of each of the treated fungal strains.

Colonization Frequency of Entomopathogenic Fungi in Different Plant Segments											
Plant Segments		Upper Part				Lower Part					
		UL		US		LL		LS		Root	
**Colonization %**	*n* *	+	%	+	%	+	%	+	%	+	%
**3 months old seedlings**											
BB Fafu-12	8	6	75	8	100	8	100	8	100	5	62.5
BB Fafu-13	8	7	87.5	7	87.5	8	100	8	100	0	0
MA Fafu-1	8	4	50	0	0	8	100	5	62.5	0	0
BB Fafu-16	8	4	50	6	75	7	87.5	7	87.5	0	0
Total	32	21	65.6	21	65.6	31	96.9	28	87.5	5	15.6
**4 months old seedlings**											
BB Fafu-12	8	5	62.5	6	75	8	100	7	87.5	3	37.5
BB Fafu-13	8	4	50	1	12.5	8	100	3	37.5	0	0
MA Fafu-1	8	4	50	0	0	6	75	0	0	0	0
BB Fafu-16	8	2	25	5	62.5	5	62.5	7	87.5	0	0
Total	32	15	46.9	12	37.5	27	84.4	17	53.1	3	9.4
**6 months old seedlings**											
BB Fafu-12	8	3	37.5	6	75	8	100	3	37.5	0	0
BB Fafu-13	8	3	37.5	0	0	4	50	3	37.5	0	0
MA Fafu-1	8	0	0	0	0	5	62.5	0	0	0	0
BB Fafu-16	8	0	0	2	25	7	87.5	1	12.5	0	0
Total	32	6	18.8	8	25	24	75	7	21.9	0	0

*n* * Total number of plants sampled per each fungal strain. + Number of plants from which fungal endophyte was re-isolated out of the 8 plants examined (at least 1 of the 5 leaf discs, stem, or root cuttings showed fungal outgrowth). **%** Colonization frequency percentage (number of plants from which endophytic fungi was re-isolated ÷ total number of plants assessed × 100). UL—upper leaves and US—upper stems; are young/newly developed (unmarked) plant parts that emerged post-inoculation. LL—lower leaves and LS—lower stems; are old (marked) plant parts that were directly sprayed with fungal conidia suspension at inoculation date. Eight different plants were sampled for systemic endophytic colonization per each fungal strain. Within each plant, upper leaves (UL), upper stems (UP), lower leaves (LL), lower stems (LS) and roots were separately assessed. Five surface-sterilized leaf discs, stem, and root cuttings were obtained per plant segments (total of 25 sections per plant). Provided fungal endophyte was recovered from one of the five discs or cuttings obtained from a single plant, the plant was assumed to have been endophytically colonized.
